# Does the dark triad predict intention to commit corrupt acts? The mediating role of financial anxiety among Saudi students

**DOI:** 10.1080/00049530.2023.2177498

**Published:** 2023-03-05

**Authors:** Radeah Mohammed Hamididin, Mogeda El Sayed El Keshky

**Affiliations:** a Department of Psychology, Faculty of Arts and Humanities, King Abdulaziz University, Jeddah, Saudi Arabia; b Department of Psychology, Faculty of Arts, Assiut University, Assiut, Egypt

**Keywords:** Machiavellianism, psychopathy, narcissism, dark triad, corruption, financial anxiety

## Abstract

**Objective:**

Corruption is a threat to the economies and overall wellbeing of nations, organizations, and individuals, and it is important to understand corruption’s antecedents and pathways through which it operates. The aim of this study was to investigate the relationship between the dark triad personality traits and corruption propensity, and to examine a mediation role of financial anxiety.

**Method:**

A sample of 699 respondents (72.5% of females, mean age = 24.3, SD = 6.65) was involved in this study. Respondents completed a survey containing demographic questions, the Dark Triad Dirty Dozen scale, the Corruption Propensity Scale, and the Financial Anxiety Scale. Structural equation models were estimated to investigate the relationships among variables.

**Results:**

The main findings indicated that only psychopathy was directly related to corruption propensity while narcissism and Machiavellianism were associated with corruption propensity only through financial anxiety. This indicates that financial anxiety fully mediated the relationship between narcissism and Machiavellianism, respectively, and corruption propensity, but did not mediate the relationship between psychopathy and corruption.

**Conclusion:**

psychopathy appears to be an important predictor of corruption propensity. In addition, financial anxiety plays an important role as a trigger for corruption propensity in narcissists and Machiavellians. Therefore, it is crucial to take financial anxiety into account when designing policy against corruption.

## Key Points

**What is already known about this topic**:

1. Corruption deteriorates people’s well-being and nations’ wellbeing.
2. Studies have shown that corruption results from dark personality traits.
3. Research has also shown some paths through which this relationship operates, including anxiety.

**What this topic adds**:

(1) This study shows that financial anxiety facilitates Machiavellians and Narcissists to engage in corruption.
(2) Psychopaths do not need to have financial anxiety to engage in corruption, they are already more prone to corruption.
(3) Programs aimed to eradicate corruption should put emphasis on psychopathy and financial anxiety in people.

## Introduction

Corruption is a malevolent behaviour that exists in all countries, affecting individuals, organizations, and societies, although not proportionally. Corruption has a negative impact on performance and reduces organizational satisfaction (Park & Blenkinsopp, 2011). Corruption also presents challenges that weaken an organizational environment (Kenny, 2007). Corruption is widespread throughout the world and has arguably been asserted as one of the ugly sides of globalization (Park, 2003). Corruption hinders economic development and alters investment flow and international trade (Glynn et al., 1997). The World Bank and the Organization for Economic Cooperation and Development identify corruption as the big challenge of the globalized economy (Park, 2003).

In some cultures, corruption results from social norms that value loyalty and gift giving over law. Thus, what is considered a bribe in one culture may be consodered a gift in another (Persson et al., 2013). The Saudi society has extended family in the centre of its social structure. Accordingly, for Saudis, bloodlines shapes social status more than wealth and success (Alshalan, 2017), which means one has to devote loyalty to the family in the first place. In addition, the code of honour governs interpersonal relationships and business transactions in Saudi Arabia, which implies that transactions that are judged dishonourable whether legal or illegal, are considered immoral (Alshalan, 2017).

Corruption is usually defined as deliberate deviant behaviour that breaks legal and/or moral societal norms and uses public or others’ resources for personal benefit (Rabl & Kühlmann, 2008). This definition implies some form of the malicious personality traits that have been conceptualized under the term “dark triad” (Paulhus & Williams, 2002). Corruption is not only an economic issue, but a question of morality (Collier, 2002), which indicates that those with immoral personality traits would be more likely to engage in corrupt behaviours. Prior research has documented that the dark triad personality traits are responsible for many unethical behaviours and misconduct (Azizli et al., 2016; Roeser et al., 2016). But, through which mechanisms would people be keen to engage in corruption, knowing it is illegal and/or unethical? Previous research has asserted the dark triad has a positive relationship with corruption intention through the path of anxiety (Hajhoseiny et al., 2019), but in this study we hypothesized the path of, specifically, financial anxiety, where those people who score high on the dark triad might engage in corruption when they are anxious about their finances.

Financial anxiety derives from lack of financial resources (Roll et al., 2016) and, consequently, it is more prevalent in low-income people. In America, for example, employees who earned lower wages were ten times more likely to report financial stress than those who were earning higher wages (Board of Governors of the Federal Reserve System, 2016; Qian, 2013). Moreover, households earning less than $50,000 reported significantly higher financial stress than those earning more than $50,000 (American Psychological Association [APA], 2015). Among students, research has shown that when they take loans for their education, this contributes significantly to their financial anxiety. It was reported in a sample of female students that financial anxiety was prevalent, especially due to student loans (Archuleta et al., 2013). In Saudi Arabia, a study found that anxiety was higher among those who were experiencing a financial burden compared to those who were not (Alshamlan et al., 2020). It is well conceivable that worry about scarcity of financial resources might trigger someone to have corrupt intentions and indeed engage in corrupt acts, especially when they have high levels of dark triad personality traits.

According to Lesnik and Blanc (1990), scarcity is the father of corruption, especially in developing countries. People do not always have enough money to support their families, which make them prone to corruption (Park, 2003). In fact, people may be willing to do anything possible, even becoming involved in criminal activities such as corruption, when they fail to meet the resources needed for survival (Staw & Szwajkowski, 1975). Similarly, Sachin et al. (2021) found that the lack of emergency assets was associated with financial anxiety. Accordingly, students who were struggling with their financial situations would experience financial anxiety which might make them engaging in corrupt intentions, especially when they have high levels of dark triad personality traits.

Previous studies have examined the relationship between the dark triad and corruption, but financial anxiety was neglected despite its potential impact. The significance of this study resides in fact that it is the first study to investigate financial anxiety to explain the relationship between the dark triad and corruption. To the best of our knowledge, no prior study has hypothesized the mediational role of financial anxiety in this relationship. Therefore, the purpose of this study was to contribute to the literature by investigating this relationship in a sample of Saudi students.

## Dark triad and corruption

The dark triad consists of three malicious personality traits: Machiavellianism, psychopathy, and narcissism (Paulhus & Williams, 2002). Machiavellianism is characterized by a high will to manipulate others in order to achieve personal goals and an absence of moral principles (Pechorro et al., 2021). Those who score high on psychopathy have traits such as impulsivity, lack of remorse, manipulativeness, lack of responsibility, and abusive charming (Hare & Neumann, 2008). Narcissism is characterized by entitlement, grandiosity, vanity, lack of empathy, and obsession with self-enhancing behaviours (Raskin & Hall, 1979; Smith & Lilienfeld, 2013). Prior metanalytic reviews has established a link between the dark triad and a range of work-related outcomes. The dark triad was related to both leadership effectiveness and managerial derailment in a review by Spain et al. (2014). O’Boyle et al. (2012) concluded in their meta-analysis that Machiavellianism, psychopathy, and narcissism were all related to counterproductive work behaviours, which might include corruption. A plausible inference would be that individuals who score high on these traits would be more likely to engage also in corruption, and previous research has indeed established these relationships. For example, Zhao et al. (2016) and Putri et al. (2021) found dark triad personality traits to positively predict corruption intent. Therefore, we formulate the first hypothesis as follows:

H1: *The dark triad personality traits are positively associated with corruption propensity*

## Dark triad and financial anxiety

The dark triad (narcissism, Machiavellianism, and psychopathy) are personality traits characterized by social aversiveness, manipulative behaviours, and other self-serving behaviours and are linked to anxiety. Previous research has established that narcissism can lead to distress and unhappiness (Fang et al., 2021; Gómez-Leal et al., 2019), which are related to anxiety. Machiavellianism has also been shown to be related to anxiety. In a sample of females, it was found that those who scored higher on Machiavellianism had increased anxiety symptoms (Sabouri et al., 2016). Studying psychopathy among violent offenders, Skeem et al. (2007) emphasized the propensity of psychopaths to anxiety. Derefinko (2015) also stated that psychopathy may be associated with anxiety. Shengbo et al. (2022) concluded that psychopaths often experience anxiety because of their lack of emotional response and lack of empathy (Shengbo et al., 2022).

Nonetheless, the dark triad traits exhibit different relationships with some outcomes that are related to anxiety. For example, narcissism exhibit a positive relationship with mental toughness and a negative relationship with emotional reactivity while Machiavellianism and psychopathy exhibit a negative relationship with mental toughness and a positive relationship with emotional reactivity to stress (Birkás et al., (2016); Onley et al., 2013). Moreover, others reported that Machiavellianism and psychopathy were positively correlated with alexithymia whereas a negative correlation was reported for narcissism (Cairncross et al., 2013). Lyons et al. (2019) found that narcissism buffered the effect of stress on depression and psychosis whereas Machiavellianism and psychopathy were directly related to anxiety. Another study showed that narcissism was positively related to mental toughness and resilience while Machiavellianism and psychopathy showed no association with mental toughness and resilience (Szabó et al., 2022). It is noteworthy to mention also that within the construct of narcissism, two aspects are differentiated and show different relationships with some variables. For example, grandiose narcissism is related to positive outcomes whereas vulnerable narcissism is related to negative outcomes (Besser & Zeigler-Hill, 2011). Parallelly, Papageorgiou et al. (2019) reported that grandiose narcissism increased mental toughness while vulnerable narcissism decreased mental toughness. Similarly, Derefinko (2015) reported mixed results concerning anxiety and psychopathy. Testing “the psychopathic low anxiety” hypothesis, they found a small relationship between anxiety and the total score of psychopathy, and while factor 1 of psychopathy was negatively related to anxiety, factor 2 was positively related to anxiety. While Kowalski et al. (2021) found a positive relationship between Machiavellianism and anxiety, no association was reported between psychopathy and anxiety. Although the dark triad exhibit different relationships with anxiety, we argue that anxiety about money or one’s financial situation is another different dimension that may be associated with the dark triad. Desperation for money can push individuals to have corruption intention if the occasion is presented, and way more for those individuals with the dark side of personality. As such, we formulate our second hypothesis as follows:

H2: *The dark triad personality traits are positively associated with financial anxiety*

## Financial anxiety as a mediator

Financial anxiety can be defined as a psychological worry and unhealthy attitude towards thinking about, engaging in, and managing personal finances effectively (Shapiro & Burchell, 2012). As such, financial anxiety can act as the path through which the dark triad impacts on corruption. Narcissists have a high sense of grandiosity and are attention seekers. Hence, they experience a gap between their undesired self and actual self (Hajhoseiny et al., 2019). Therefore, narcissists suffer with self-esteem issues, which has been shown to be related to anxiety (Bajaj et al., 2016). Machiavellians score low in emotional intelligence (Tsirimokou et al., 2021), and it was reported that individuals with low emotional intelligence lack the ability to express emotions, and consequently are unable to effectively manage stressful situations (Schutte et al., 2001). This indicates that Machiavellians can be prone to anxiety, as indeed has been reported by Kubak and Salekin (2009). Individuals high on Machiavellianism experience emotional instability, and cannot express their emotions as delicately as their as their counterparts (Szijjarto & Bereczkei, 2015). It is also noteworthy to note that others argued that Machiavellians manage stressful situations through Machiavellian intelligence, which gives them ability to adapt in any social context (Bereczkei, 2018). Psychopaths lack remorse and guilt. However, previous studies have shown that psychopaths experience anxiety because of their antisocial behaviours and the risks they take (Sandvik et al., 2015). Anxiety can lead to the mobilization of various kinds of psychological and physiological resources to escape or avoid danger (Rachman, 2004) and triggers psychological, physiological, and behavioural resources in order to counter the unpleasant feelings (Pacheco-Unguetti et al., 2010). Thus, seeking to “remedy” their emotional distress, individuals may adopt ideas and behaviours that include corrupt intent and acts. The Affective Events Theory (Weiss & Cropanzano, 1996) postulates that the emotional state of employees impacts workplace attitudes and behaviours, which can lead to unethical behaviours including corruption. The mediational role of financial anxiety can be explained by the Conservation of Resources theory (Hobfoll, 1989) since anxiety can act as a draining resource, and acquiring money, in any way, would compensate the loss (Hajhoseiny et al., 2019).

H3: *Financial anxiety positively mediates the relationship between the dark triad personality traits and corruption propensity*

**Figure 1.**
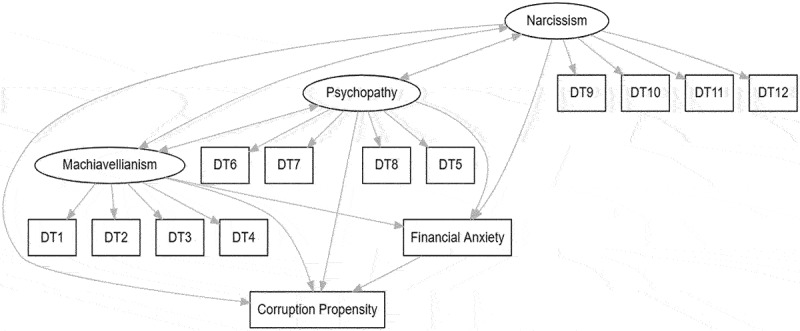
Contextual model.

*Note*: DT*n* are the numbered items in the Dark Triad Dirty Dozen scale.

The conceptual model is summarized in Figure 1. The dark triad traits are hypothesized to have positive relationships with corruption propensity, and these relationships are hypothesized to be mediated by financial anxiety.

## Methods

### Participants

This study used a convenience sample of university students in order to reach as many and diverse participants as possible. A link to the survey was sent via emails, Facebook, WhatsApp, and Twitter. A total of 699 respondents returned the completed survey. They were informed about the aim and the intended outcome of the study, and they provided informed consent. The sample included 505 females (72.5%) and 192 males (27.5%). The sample had a mean age of 24.3 (SD = 6.65), ranging between 18 and 51. Other socio-demographic characteristics of the sample are as follows: Around 77.5% were single, 16.3% were married, 5.6% were divorced, and 0.6% were widowed. Around 21.6% earned less than 5000 RS, 22.1% earned at least 5000 RS and less than SR 8000, 16.2% earned between 8000 RS and less than SR 11,000, and 40.1% were earning 11,000 SR or more.

### Measures

This study used three assessment scales: The Dark Triad Dirty Dozen (Jonason & Webster, 2010), the Corruption Propensity Scale (Agbo & Iwundu, 2015), and the Financial Anxiety Scale (Archuleta et al., 2013). Along with these scales, the questionnaire included demographic characteristics including gender, age, education, marital status, income, nationality, and job type.

The Dark Triad Dirty Dozen (Jonason & Webster, 2010) is a twelve-item instrument that measures Machiavellianism, psychopathy, and narcissism, with 4 items for each trait. Respondents are asked to rate how strongly they agree with the statements on a 9-point Likert scale from 1 (not at all) to 9 (very much). Therefore, total scores for each respective trait range from 4 to 36. The scale includes items like “I tend to exploit others towards my own end” for Machiavellianism, “I tend to lack remorse” for psychopathy, and “I tend to want others to admire me” for narcissism. The scale exhibited acceptable internal consistency reliability in previous studies (Machiavellianism α = 0.69, psychopathy α = 0.65, and narcissism α = 0.81). In this study, the internal consistency reliability was adequate except for psychopathy (Machiavellianism α = 0.78, psychopathy α = 0.58, and narcissism α = 0.85).

The Corruption Propensity Scale (Agbo & Iwundu, 2015) is a 18-item instrument designed to measure corruption intent. The scale is scored on a 7-point Likert scale ranging from 1 (strongly disagree) to 7 (strongly agree). Thus, total possible scores range between 18 and 126. Higher scores indicate greater propensity to engage in corruption. The scale includes items like “I do not mind favouring a client for a bribe” and “If I have the opportunity of handling any money that belongs to this country, I will make sure I benefit from it”. The scale exhibited an adequate internal consistency reliability (Cronbach’s alpha = 0.91). In this study, the Cronbach’s alpha was 0.90.

The Financial Anxiety Scale (Archuleta et al., 2013) is a 7-item scale that is used to assess an individual’s financial anxiety. The scale is scored on a 7-point Likert scale, ranging from 1 (never) to 7 (always). Possible scores range between 7 and 49. Higher scores indicate greater financial anxiety. The scale includes items like “I feel anxious about my financial situation” and “I feel fatigued because I worry about my financial situation”. The scale was found to have excellent internal consistency reliability (Cronbach’s alpha = 0.94) (Archuleta et al., 2013). In this study, the Cronbach’s alpha was 0.92, indicating excellent internal consistency reliability for the scale.

### Procedure

This study used a cross-sectional design and was conducted online. All procedures performed in this study were in line with the ethical standards of King Abdulaziz University and the 1964 Helsinki Declaration and its later amendments or comparable ethical standards. The respondents completed the demographic questionnaire first, and then completed the Dark Triad Dirty Dozen questionnaire, the Corruption Propensity Scale, and the Financial Anxiety Scale. Before beginning, respondents were given instructions and only upon consent could they move on to the questionnaires.

### Statistical analysis

All the statistical analyses were carried out in RStudio (Ihaka & Gentleman, 1996). Descriptive statistics and ANOVA tests were computed first. Second, Pearson correlation coefficients were calculated, followed by Cronbach’s alphas, which were computed using the “psych” statistical software package (Revelle, 2017). Third, structural equation models were used to estimate the mediation analysis, using the “lavaan” package (Rosseel, 2012). To compare different models, various fit indices were used, including chi-square, SRMR, RMSEA, TLI, and CFI (Hu & Bentler, 1999). The figures were plotted using the “lavaanPlot” package (Lishinski, 2020).

## Results

The results of the socio-demographic characteristics of the sample and the results of the ANOVAs are summarized in Table 1.

**Table 1.** Descriptive statistics of the sample and ANOVA tests.

Variable	n	%	Mean (SD) Corruption
Gender			*p = 0.886*
Male	192	27.5	38.5 (19.6)
Female	507	72.5	38.3 (15.6)
Academic level			*p = 0.05*
1	207	29.6	40.7 (16.2)
2	170	24.3	37.8 (15.1)
3	322	46.1	37.2 (17.9)
Marital status			*p<0.05*
Single	542	77.5	38.6 (15.6)
Married	114	16.3	36.4 (22.3)
Divorced	39	5.6	42.4 (13.3)
Widowed	4	0.6	21 (3.4)
Mother’s education			*p<0.05*
Uneducated	93	13.3	41.8 (14.9)
Less than high school	119	17	35.5 (12.9)
High school	260	37.2	39.6 (19.3)
University degree	178	25.5	36.9 (14.1)
Master’s degree/PhD	49	7	37 (21.7)
Father’s education			*p<0.001*
Uneducated	45	6.4	*40.8 (14.9)*
Less than high school	98	14	*39.9 (14.9)*
High school	341	48.8	*37.5 (15.8)*
University degree	176	25.2	*37.4 (15.3)*
Master’s degree/PhD	39	5.6	*50.2 (29.5)*
Parental marital status			*p = 0.109*
Parents are deceased	10	1.4	25 (3.1)
Divorced	84	12	36.9 (16)
Parents live together	533	76.4	*38.6 (16.9)*
Mother is widowed	58	8.3	39.4 (18.9)
Father is widowed	14	2	40.7 (10.7)
Income (per month)			*p = 0.363*
Less than 5000RS	151	21.6	36 (16.9)
5000 - less than SR 8000	155	22.1	41.3 (19.1)
8000 - less than SR 11,000	113	16.2	38.2 (11.3)
11000 SR and more	280	40.1	37.9 (17.5)

The descriptive statistics for the main study variables are summarized in Table 2. The mean score was 8.43 (SD = 5.64, range = 4–36) for Machiavellianism, 9.56 (SD = 5.17, range = 4–36) for psychopathy, 20.06 (SD = 8.65, range = 4–36) for narcissism, 17.1 (SD = 10.2, range = 7–49) for financial anxiety, and 38.3 (SD = 16.8, range = 18–126) for corruption propensity.

**Table 2.** Mean, SD, skewness, kurtosis, and Cronbach’s alpha.

Variable	Mean	SD	Range	Skewness	Kurtosis	α
1. Machiavellianism	8.43	5.64	4–36	1.71	3.25	0.78
2. Psychopathy	9.56	5.17	4–36	1.06	1.09	0.58
3. Narcissism	20.06	8.65	4–36	−0.01	−0.85	0.85
4. Financial anxiety	17.1	10.2	7–49	1.21	0.72	0.92
5. Corruption	38.3	16.8	18–126	1.48	3.27	0.90

The bivariate correlations between the variables are displayed in Table 3. Corruption was positively correlated with Machiavellianism (r = 0.37, p < 0.001), with psychopathy (r = 0.41, p < 0.001), with narcissism (r = 0.20, p < 0.001), and with financial anxiety (r = 0.23, p < 0.001). Financial anxiety was positively correlated with Machiavellianism (r = 0.15, p < 0.001), with psychopathy (r = 0.11, p < 0.001), and with narcissism (r = 0.25, *p *< 0.001).

**Table 3.** Bivariate correlations.

Variable	1	2	3	4	5
1. Machiavellianism	1				
2. Psychopathy	0.41***	1			
3. Narcissism	0.29***	0.17***	1		
4. Financial anxiety	0.15***	0.11***	0.25***	1	
5. Corruption	0.37***	0.41***	0.20***	0.23***	1

Column heading numbers correspond to the numbered variables in the row headings.

**Figure 2.**
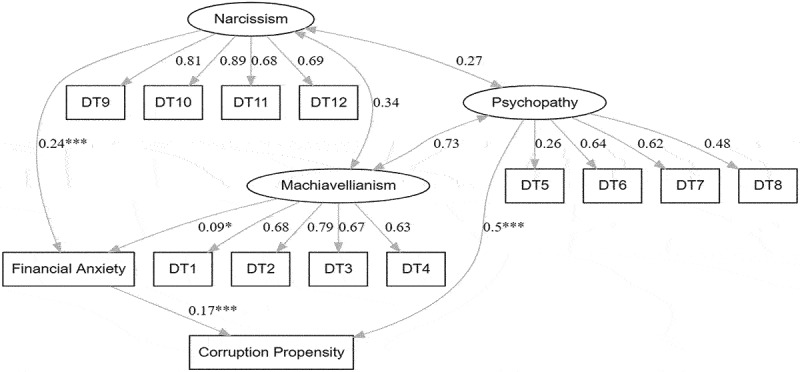
Final SEM model.

*Note*: * p < 0.05; *** p < 0.001. The numbers on the arrows represent the standardized regression coefficients, and the numbers on the double-sided arrows represent the covariance.

For the mediation analysis, two models of structural equation models were run. The first model estimated the dark triad traits as predictors of corruption propensity directly and indirectly through financial anxiety. This model had acceptable fit indices (*X*^2^ = 367.16, *p* < 0.001; RMSEA = 0.07; SRMR = 0.05; CFI = 0.90; TLI = 0.97). in this mode, the explained variance was 23%. However, the direct paths from narcissism to corruption and from Machiavellianism to corruption, and the path from psychopathy to financial anxiety were not statistically significant. This means that narcissism and Machiave-llianism did not directly predict corruption, however they did so through financial anxiety; financial anxiety did not mediate the relationship between psychopathy and corruption; and psychopathy predicted corruption directly. Consequently, these paths were deleted in the second model in order to improve the fit indices. Model 2 exhibited better fit indices (*X*^2^ = 3.72.23, *p* < 0.001; RMSEA = 0.05; SRMR = 0.3; CFI = 0.92; TLI = 0.90). In this model, only psychopathy directly predicted corruption (β = 0.50, *p* < 0.001), Machiavellianism predicted corruption only through financial anxiety (β_ind_ = 0.20, *p* < 0.05), and narcissism also predicted corruption through financial anxiety (β_ind_ = 0.34, *p* < 0.001). In this final model, the explained variance was 32%. This final model is shown in Figure 2.

## Discussion

Corruption is a socio-political problem that is detrimental to the wellbeing of people, organizations, and nations, and it is therefore important to examine the antecedents and pathways of corruption. The aim of this study was to investigate the relationship between the dark triad and corruption and to investigate a possible mediation role of financial anxiety. The main results indicated that psychopathy was associated with corruption propensity, but narcissism and Machiavellianism were not directly associated with corruption propensity. Thus, hypothesis 1 was partially supported. Only narcissism and Machiavellianism were related to financial anxiety. Hypothesis 2 was also partially supported. Financial anxiety fully mediated the relationships of both Machiavellianism and narcissism with corruption propensity. Hypothesis 3 was also partially supported.

The results of this study partly corroborate previous studies. In this study, only psychopathy was directly related to corruption propensity. However, Zhao et al. (2016) found that all three dimensions of the dark triad positively predicted intentions to corrupt. This was also found in a sample of Indonesian state civil servants, where dark triad was reported to be related to corruption (Putri et al., 2021). Related results were reported by Azizli et al. (2016), where only psychopathy significantly supported the prediction of misconduct behaviours. Although narcissists and Machiavellians use manipulative and exploitative strategies (Birkás et al., 2015; Emmons, 1987), psychopaths are the only ones who do not feel guilt or remorse for their unethical behaviours or for hurting others (Harrison et al., 2018). This might explain why only psychopathy was directly related to corruption propensity.

Previous studies have shown that Machiavellians, with their manipulativeness and exploitative tendencies, are likely to engage in unethical behaviour (Birkás et al., 2015; Gunnthorsdottir et al., 2002). Narcissists on the other hand have a high sense of grandiosity and are also willing to exploit others, which makes them susceptible to engaging in unethical behaviours (Duchon & Drake, 2009; Johnson et al., 2013), including corruption. However, results of this study suggested that Machiavellians and narcissists would not engage in corruption unless they have high levels of financial anxiety. This means that financial anxiety would push Machiavellians and narcissists to have corruption intentions. Examining the importance of the dark triad on corruption beyond the contribution of the HEXACO model, (Szabó et al., 2021) reported that Machiavellianism and psychopathy were related to corruption intention whereas narcissism was unrelated to corruption.

Financial anxiety fully mediated the relationship between narcissism and corruption and between Machiavellianism and corruption. This is in line with previous research that reported that the association between dark triad personality traits and corruption was fully mediated by anxiety (Hajhoseiny et al., 2019). Machiavellians are typically manipulative and exploitative in order to reach their goals (Gunnthorsdottir et al., 2002), and when they happen to experience financial anxiety, this drives them to engage in corrupt behaviours. It has been argued that Machiavellians can perform hostile behaviours when they have something special in mind to achieve (Wu & Lebreton, 2011). It seems that when narcissists are financially anxious, they can engage in corrupt behaviours. Others had reported that narcissists can engage in corrupt behaviours when their boredom is high (Gu et al., 2021). It has been postulated that individuals scoring high on narcissism tend to be self-centred and can be eager to seek profits at the price of others (DuBrin, 2012). We argue that for individuals who are manipulative in nature and have a sense of superiority to others, when they are desperate for money, they can do anything possible including corruption in order to compensate for the desperation. The relationship between psychopathy and corruption was not mediated by financial anxiety, thus it seems that psychopaths do not have to be financially anxious to engage in corruption. This is rooted in their inability to empathize and to feel guilt or remorse (Harrison et al., 2018). In previous research, psychopathy was claimed as an explanation for socially malicious behaviours such as internet fraud, embezzlement, and mortgage fraud (Babiak et al., 2010). Glenn et al. (2010) went further and reported that psychopaths engage in unethical behaviours because they do not have moral principles in their sense of identity.

This study has some limitations that must be acknowledged. First, this study used a cross-sectional design and we cannot conclude any causality or direction of the relationships. Future research should use longitudinal designs. Second, the sample was determined using convenience sampling methods and random sampling should be used in future research. Third, this study used self-reported measures. Objective measures should also be used in future research. Fourth, this study’s sample was disproportionate, with more than 72% of respondents being females, and future research should use a more proportionate sample. Further, the correlations between the variables were rather small, and the psychopathy subscale had a relatively small internal consistency reliability coefficient.

## Conclusion

This study investigated the relationship between the dark triad and corruption intent and the mediation role of financial anxiety among a sample of students in Saudi Arabia. Psychopathy was associated with corruption, and narcissism and Machiavellianism were associated with corruption only through financial anxiety. In other words, in the absence of financial anxiety, only those students who scored high on psychopathy exhibited corrupt intentions. It is important to understand the antecedents and pathways through which corruption operates in order to better inform proper policy against it. Psychopathy seems to be an important antecedent of corruption propensity, and financial anxiety should be taken into account when designing programs and policy against corruption.

## Data Availability

The data are available from the corresponding author, upon reasonable request.
